# Multi-method Machine-Learning Identification of IL11 as a Key Gene in Kidney Stone Disease: Mechanistic Clues and Diagnostic Potential

**DOI:** 10.7759/cureus.116828

**Published:** 2026-09-24

**Authors:** Wenlong Yu, Lei Xie, Jiajie Zhang, Yongqiang Liang

**Affiliations:** 1 Urology, Guilin Hospital of Traditional Chinese Medicine, Guilin, CHN

**Keywords:** diagnostic nomogram, gsea, il11, immune infiltration, kidney stones, machine learning, randall’s plaque, wgcna

## Abstract

Background: Randall’s plaque (RP) is a key initiating lesion of idiopathic calcium stones at the renal papilla, and transcriptomic profiling of human RP has revealed inflammation- and matrix-remodeling-related changes, including upregulation of interleukin 11 (IL11). However, under a pure kidney-stone phenotype, a rigorous framework combining multi-algorithm consensus screening with fuller diagnostic appraisal (calibration, internal validation, and decision-curve analysis (DCA)) remains needed.

Methods: GSE73680 (RP, n=29; normal papilla, n=33) was obtained from the Gene Expression Omnibus (GEO). Exploratory differential expression (limma) and weighted gene co-expression network analysis (WGCNA) were intersected, followed by Gene Ontology (GO)/Kyoto Encyclopedia of Genes and Genomes (KEGG) enrichment and Search Tool for the Retrieval of Interacting Genes/Proteins (STRING) protein-protein interaction (PPI) analysis. Consensus genes were defined by five feature-selection algorithms: least absolute shrinkage and selection operator (LASSO), support vector machine recursive feature elimination (SVM-RFE), random forest, XGBoost, and Boruta. For IL11, we assessed expression, built a penalized logistic nomogram with receiver operating characteristic (ROC), calibration, and patient-blocked internal validation, and performed Cell-type Identification by Estimating Relative Subsets of RNA Transcripts (CIBERSORT) deconvolution and correlation-ranked KEGG gene set enrichment analysis (GSEA).

Results: The intersection of 39 exploratory differentially expressed genes (DEGs) with the WGCNA green module yielded 23 candidates, of which IL11 was the sole five-method consensus gene and was significantly upregulated in RP (Wilcoxon P=8.9×10⁻⁵). The single-gene model achieved an apparent area under the curve (AUC) of 0.782 (95% confidence interval (CI) 0.663-0.901) and an out-of-bag (OOB) AUC of 0.756. PPI linked IL11 to SPP1 and TNFRSF11B, and GSEA enriched lysosome, cell adhesion molecules, and leukocyte transendothelial migration pathways.

Conclusions: Building on prior studies, this work strengthens the evidence chain for IL11 as a priority target under a pure kidney-stone/RP phenotype through five-algorithm consensus, complete diagnostic appraisal, and adhesion/migration-related mechanistic clues.

## Introduction

Kidney stone disease is a common urological disorder, and calcium oxalate stones account for the majority of cases [1]. Idiopathic hypercalciuria and papillary mineralization constitute the major pathophysiological background of calcium stone formation [2]. Randall’s plaque (RP) is a critical initiating nidus on which idiopathic calcium oxalate stones attach and grow; plaque begins in basement membranes of the thin loops of Henle and expands into the papillary interstitium [3]. Immunity and inflammation contribute substantially to crystal nephropathy, with macrophage polarization and matrix remodeling repeatedly documented in calcium oxalate stone disease [4,5]. Identifying reproducible molecular markers from human RP transcriptomes therefore has direct translational relevance.

Taguchi et al. established the human RP microarray dataset GSE73680 and reported upregulation of LCN2, interleukin 11 (IL11), PTGS1, GPX3, and MMD, together with proinflammatory transcriptional networks [6]. Using the same main contrast of 29 RP versus 33 normal papillae, Hong et al. highlighted SPP1 as a network hub associated with macrophage infiltration [7]. Yu et al. combined weighted gene co-expression network analysis (WGCNA) with immune infiltration and focused on DCN, LUM, and P4HA2 [8], and a further re-analysis of the same resource examined oxidative-stress gene sets in RP [9].

Within kidney stone research, Shen et al. identified IL11 as a shared candidate biomarker for kidney stones and diabetes using least absolute shrinkage and selection operator (LASSO), random forest, and support vector machine recursive feature elimination (SVM-RFE) [10]. Li et al. prioritized IL11 through differentially expressed gene/protein-protein interaction (DEG/PPI) analyses and reported elevated serum IL11 as an independent associated factor in idiopathic calcium oxalate stone disease [11]. A recent review also placed IL11 within the interleukin-chemokine network of calcium oxalate nephrolithiasis [12]. IL11 is therefore not an unexplored target; what remains limited is an integrative analysis restricted to a pure kidney-stone/RP phenotype, starting from DEG∩WGCNA filtering, applying five feature-selection algorithms, and jointly reporting nomogram, calibration, internal validation, and decision-curve analysis (DCA). Here we used GSE73680 to evaluate the robustness, diagnostic potential, and mechanistic clues of IL11 and to compare our design with these published studies.

## Materials and methods

Data source and grouping

The study was conducted at the Guilin Hospital of Traditional Chinese Medicine, Guilin, China. Expression profiles were obtained from the Gene Expression Omnibus (GEO) dataset GSE73680 (platform GPL17077) [6]. Grouping followed the main contrast used by Hong et al. [7]: Case, RP tissues from calcium stone formers (n=29); Control, normal papillary tissues from stone formers plus non-stone controls (n=33). The Series Matrix was processed with probe annotation and gene-level summarization for downstream analyses.

Differential expression and WGCNA

Differential expression was analyzed with limma. Under the strict threshold of false discovery rate (FDR) <0.05 and *|log2 fold change (FC)| >1*, no significant genes were detected. To enable intersection with co-expression modules and machine-learning input, an exploratory threshold of P<0.05 and *|log2FC| >1* was used; volcano plots were drawn with raw P values to match this selection logic. For WGCNA, the top 5,000 genes by median absolute deviation (MAD) were used to construct an unsigned network; soft-thresholding power was selected by the scale-free criterion, and trait-related module genes were extracted.

Overlapping genes, functional enrichment, and PPI

Exploratory DEGs were intersected with trait-related module genes. Gene Ontology (GO) and Kyoto Encyclopedia of Genes and Genomes (KEGG) enrichment analyses were performed with clusterProfiler. PPI networks were constructed in the Search Tool for the Retrieval of Interacting Genes/Proteins (STRING) (Homo sapiens, combined score ≥0.4).

Machine-learning feature selection

Overlapping genes were used as candidate features for LASSO, SVM-RFE, random forest, XGBoost, and Boruta. Combined use of LASSO, random forest, and SVM-RFE has previously been applied in stone-related transcriptomic biomarker studies [10]. The intersection across the five methods defined consensus genes.

Expression validation, diagnostic modeling, immune infiltration, and gene set enrichment analysis (GSEA)

IL11 expression was compared between Case and Control by the Wilcoxon rank-sum test. A penalized logistic model with IL11 as the sole predictor was used to construct a nomogram; receiver operating characteristic (ROC), calibration, bootstrap/patient-blocked internal validation, and DCA were reported. Immune-cell fractions were estimated by Cell-type Identification by Estimating Relative Subsets of RNA Transcripts (CIBERSORT) (LM22, quantile normalization=TRUE, permutations=1000), and Spearman correlations between IL11 and cell fractions were calculated. For mechanistic analysis, genome-wide Spearman correlations with IL11 were used as the ranking metric for KEGG GSEA against Molecular Signatures Database (MSigDB) gene sets (c2.cp.kegg.v7.4).

## Results

Differential expression analysis

After completion of preprocessing and gene-level summarization, the final expression matrix comprised 32,080 genes measured across 62 samples. Using the exploratory threshold defined above (P<0.05 and *|log2FC| >1)*, a total of 39 DEGs were identified, of which 32 were upregulated and seven were downregulated in RP tissue; notably, IL11, PTGS1, and SPP1 were among the upregulated genes, a direction of change fully consistent with the RP upregulation pattern originally reported by Taguchi et al. in the same dataset [6]. As anticipated from the analytical design, no genes remained significant at FDR<0.05, indicating that the transcriptomic signal separating RP from normal papilla is relatively modest in this microarray cohort; exploratory DEGs were therefore used for the subsequent intersection analyses, and this methodological choice is explicitly stated here to ensure full transparency and reproducibility. Figure 1 visually summarizes these results through the volcano plot of differential expression and the corresponding expression density heatmap across samples.

**Figure 1:**
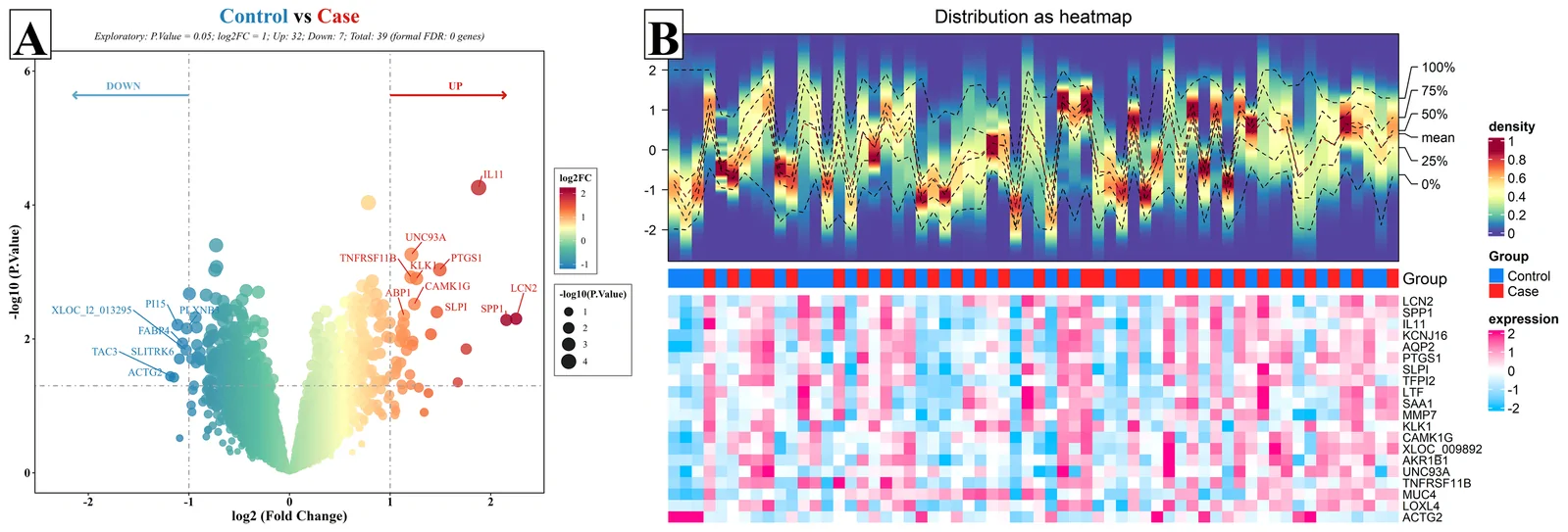
Differential expression analysis. (A) Volcano plot generated from the limma differential expression analysis, in which the x-axis shows the log2 fold change (FC) and the y-axis shows −log10(raw P-value); vertical and horizontal dashed lines mark the exploratory thresholds of *|log2FC| =1* and P=0.05, respectively, and representative genes of interest, including IL11, PTGS1, and SPP1, are individually labeled. (B) Expression density heatmap summarizing the distribution of expression values across all samples, providing a global overview of between-sample consistency.

WGCNA identifies a trait-related co-expression module

An unsigned co-expression network was constructed from the MAD top 5,000 genes, with the soft-thresholding power set to 9 in order to satisfy the scale-free topology criterion (Figure 2).

**Figure 2:**
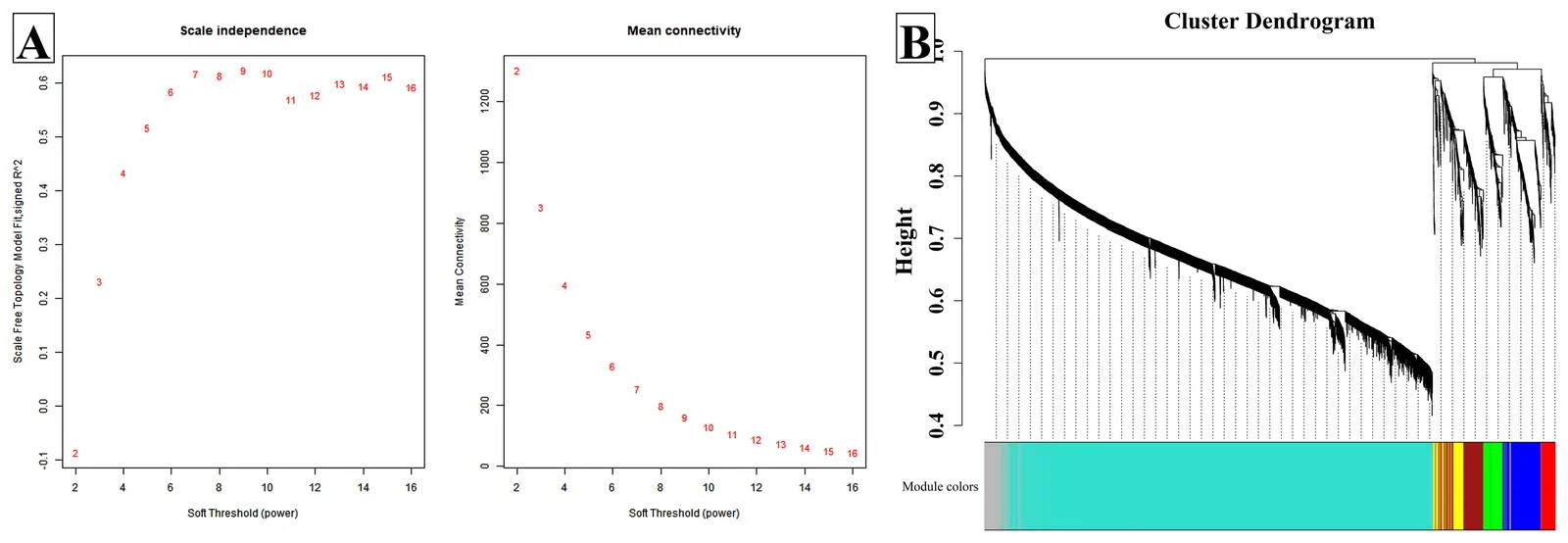
WGCNA network construction. (A) Soft-threshold selection diagnostics, including scale-free topology fit and mean connectivity across candidate powers, supporting the choice of power=9. (B) Hierarchical clustering dendrogram of genes with assigned module colors displayed underneath. WGCNA: weighted gene co-expression network analysis

Module-trait relationship analysis showed that, among all identified modules, the green module displayed the strongest association with disease grouping (approximately r≈0.26, P≈0.045); biologically, this module contained several genes of established relevance to papillary physiology and stone biology, including SPP1 and AQP2, supporting its interpretability as a disease-related module. The magnitude of this module-trait correlation was comparable to that reported for trait-related modules by Hong et al. in their independent analysis of the same dataset [7], supporting the inference that the co-expression structure reconstructed here meaningfully captures RP-related transcriptional programs rather than random expression noise (Figure 3).

**Figure 3:**
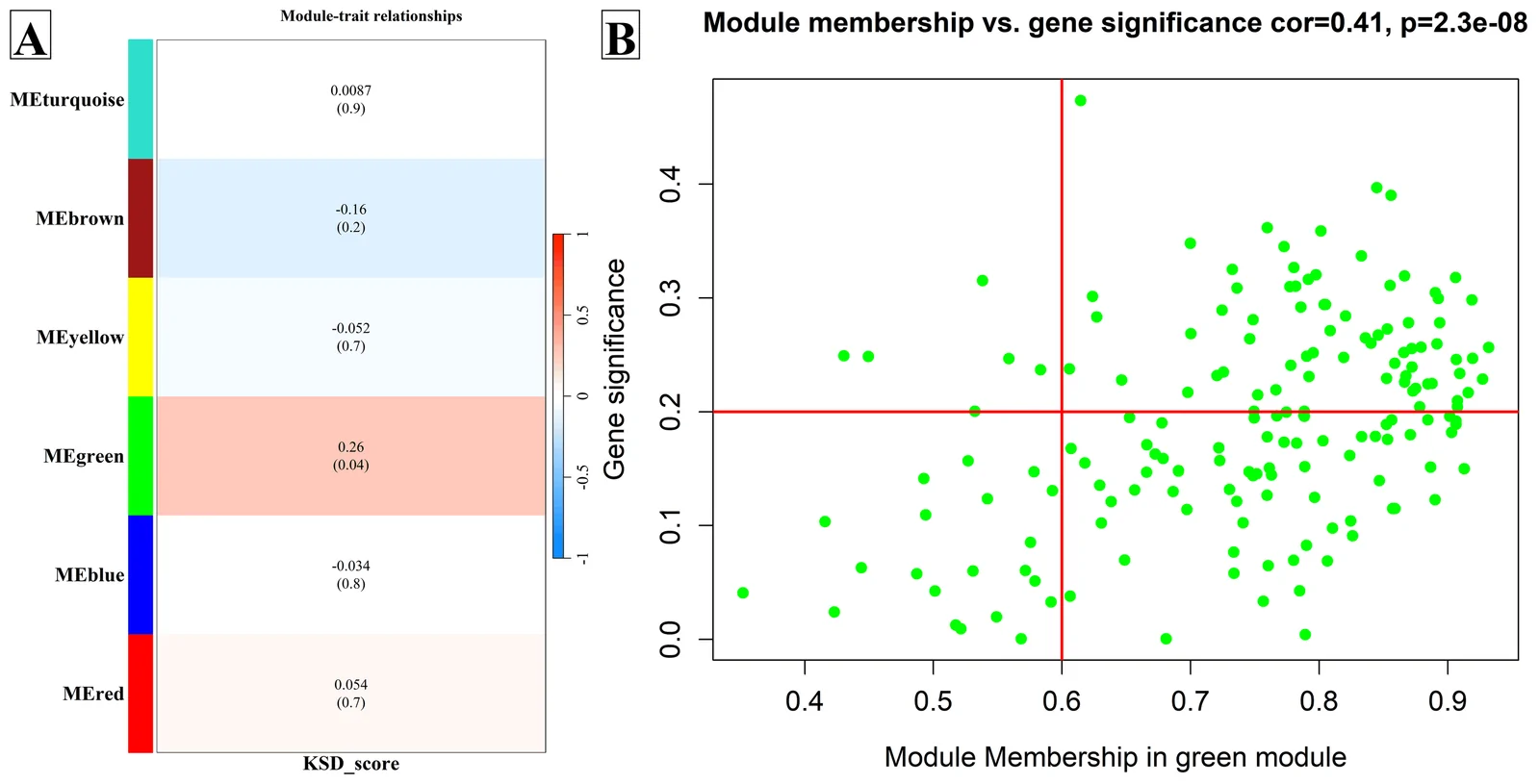
WGCNA module-trait relationships. (A) Module-trait correlation heatmap relating each module to the clinical grouping; the green module, which showed the strongest association with disease status, was selected for downstream analysis. (B) Scatter plot of gene significance (GS) versus module membership (MM) within the green module, illustrating the internal coherence of the selected module. WGCNA: weighted gene co-expression network analysis

Candidate-gene overlap, enrichment, and PPI network

Intersection of the exploratory DEGs (n=39) with the green module genes (n=172) yielded 23 overlapping genes, which were regarded as the biologically prioritized candidate pool for all subsequent analyses: ABP1, AKR1B1, AQP2, CAMK1G, DDIT4L, DPP4, FLJ44087, GCNT3, HSD11B1, IL11, KCNJ16, LOXL4, PTGS1, SFTA2, SLC14A2, SLCO4A1, SLPI, SPP1, TFPI2, TNFRSF11B, XLOC_009892, XLOC_009893, and XLOC_012102. Functional annotation showed that GO enrichment mainly involved collecting-duct development, renal water homeostasis, and basolateral plasma membrane terms, indicating that the candidate set retains a strong papillary and tubular physiological signature; KEGG analysis suggested nominal enrichment of metabolic pathways together with cytokine-cytokine receptor interaction, a pattern consistent with the matrix-inflammation background that characterizes RP pathogenesis [4,6]. At the protein-interaction level, STRING PPI analysis (combined score≥0.4) retained six edges connecting eight nodes; among these, IL11-SPP1 (score=0.499), IL11-TNFRSF11B (0.484), and TNFRSF11B-SPP1 (0.470) formed a small interconnected core, thereby directly linking IL11 to the previously emphasized SPP1 axis through protein-level association evidence (Figures 4, 5, Table 1) [7].

**Figure 4:**
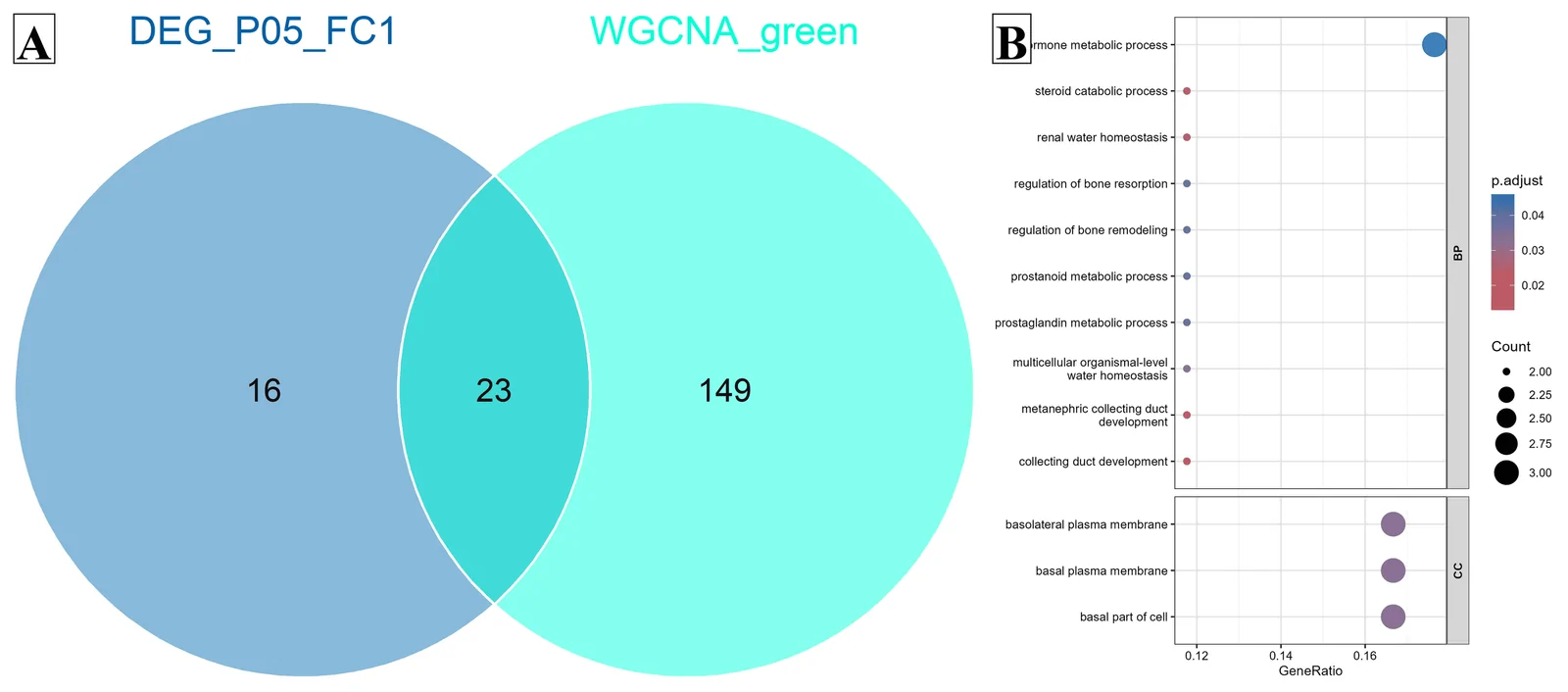
Candidate-gene overlap and GO enrichment. (A) Venn diagram showing the intersection between exploratory DEGs and the WGCNA green module, which yielded 23 overlapping candidate genes. (B) GO enrichment dot plot summarizing significantly enriched terms across the biological process (BP), cellular component (CC), and molecular function (MF) categories. DEG: differentially expressed genes; WGCNA: weighted gene co-expression network analysis; GO: Gene Ontology

**Figure 5:**
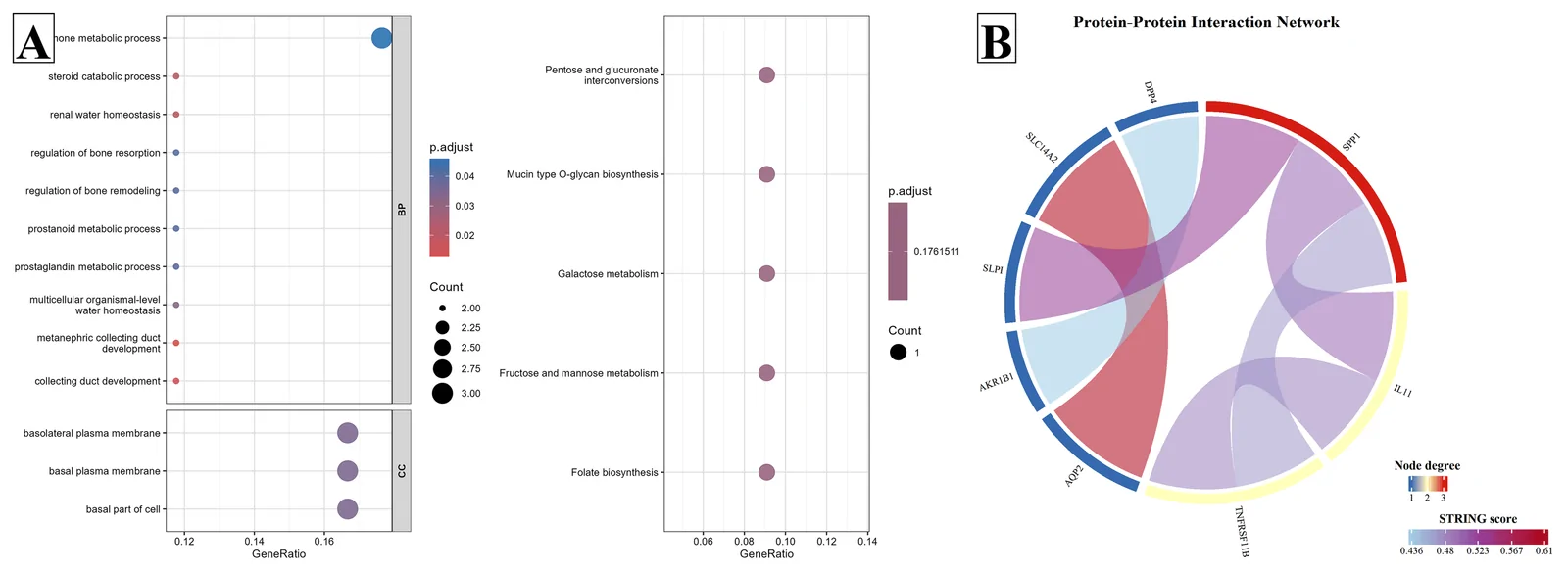
Combined enrichment and PPI network. (A) Combined GO/KEGG enrichment display integrating the results of both annotation systems for the overlapping gene set. (B) STRING PPI chord diagram of the retained interaction network, highlighting the IL11-SPP1-TNFRSF11B interconnected core. GO: Gene Ontology; KEGG: Kyoto Encyclopedia of Genes and Genomes; STRING: Search Tool for the Retrieval of Interacting Genes/Proteins; PPI: protein-protein interaction

**Table 1:** STRING PPI edges retained at combined score≥0.4. Six interactions connecting eight candidate proteins were retained, ranked here by STRING combined confidence score: AQP2-SLC14A2, 0.610; SLPI-SPP1, 0.528; IL11-SPP1, 0.499; IL11-TNFRSF11B, 0.484; TNFRSF11B-SPP1, 0.470; AKR1B1-DPP4, 0.436. STRING: Search Tool for the Retrieval of Interacting Genes/Proteins; PPI: protein-protein interaction

Protein_A	Protein_B	STRING_score
AQP2	SLC14A2	0.610
SLPI	SPP1	0.528
IL11	SPP1	0.499
IL11	TNFRSF11B	0.484
TNFRSF11B	SPP1	0.470
AKR1B1	DPP4	0.436

Five machine-learning methods converge on IL11

When the 23 overlapping genes were used as the input feature set, the five algorithms produced partially overlapping but non-identical outputs: LASSO selected IL11 and PTGS1; SVM-RFE selected IL11 as its top-ranked feature; random forest and XGBoost both ranked IL11 among their most important features; and Boruta confirmed IL11, together with several other features, as statistically important relative to shadow attributes. Strikingly, the intersection across all five methods identified IL11 as the sole consensus gene (5/5), meaning that IL11 was the only candidate independently endorsed by every algorithm applied. This convergence agrees in direction with the findings of Shen et al., who prioritized IL11 using three algorithms within a stone-diabetes comorbidity framework [10]; however, two methodological distinctions strengthen the present result: our candidate pool was derived from stone-phenotype DEG∩WGCNA filtering rather than from stone-diabetes shared genes, and the algorithm set was expanded from three to five conceptually distinct methods, yielding a stricter and more conservative consensus criterion (Figures 6, 7).

**Figure 6:**
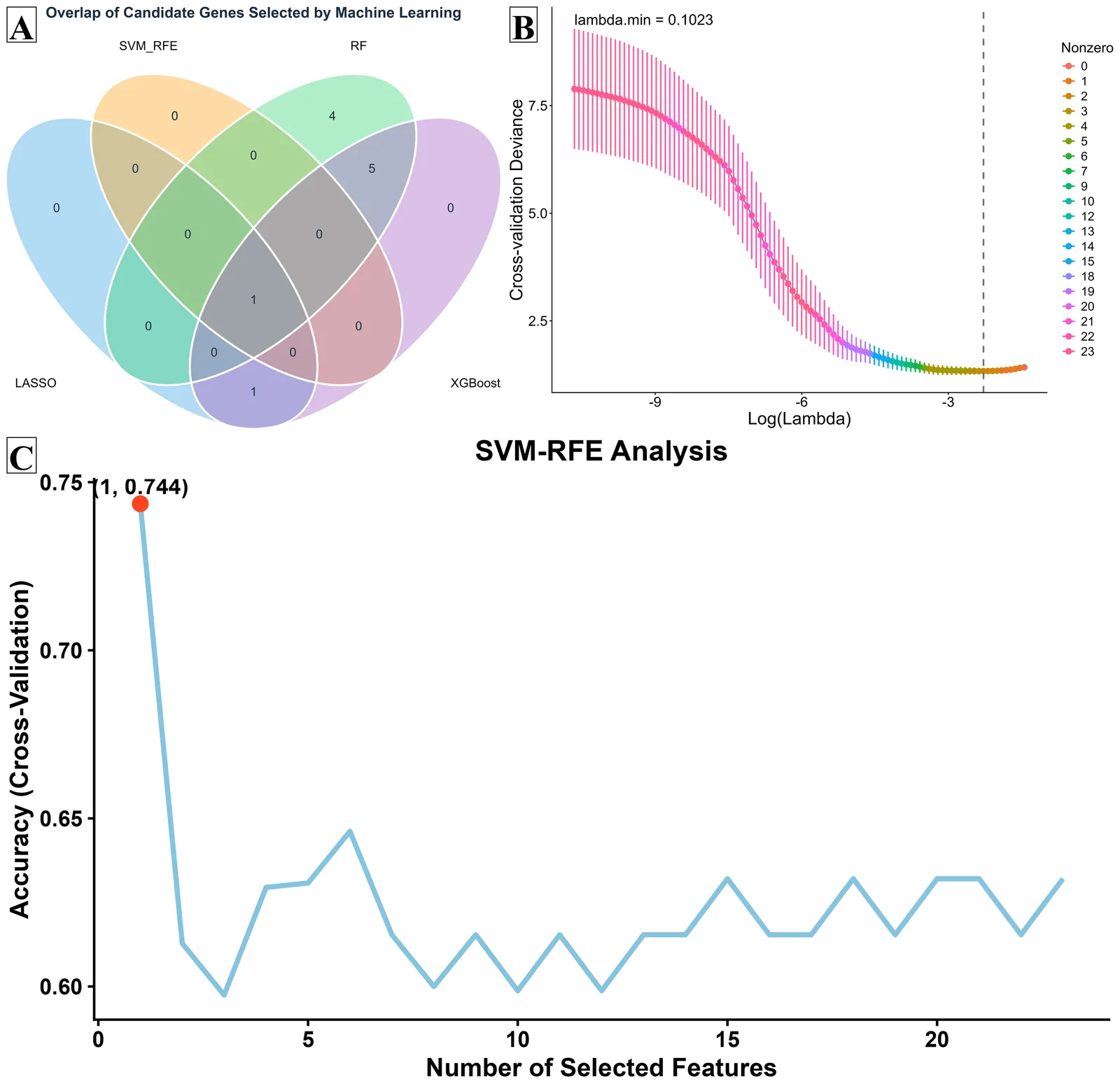
Machine-learning consensus and regularized/SVM selection. (A) Five-method Venn diagram showing the overlap of genes selected by LASSO, SVM-RFE, random forest, XGBoost, and Boruta, with IL11 located at the center intersection shared by all five methods. (B) LASSO cross-validation curve used to determine the optimal regularization parameter. (C) SVM-RFE feature-elimination curve illustrating classification performance across recursive feature subsets. LASSO: least absolute shrinkage and selection operator; SVM-RFE: support vector machine recursive feature elimination

**Figure 7:**
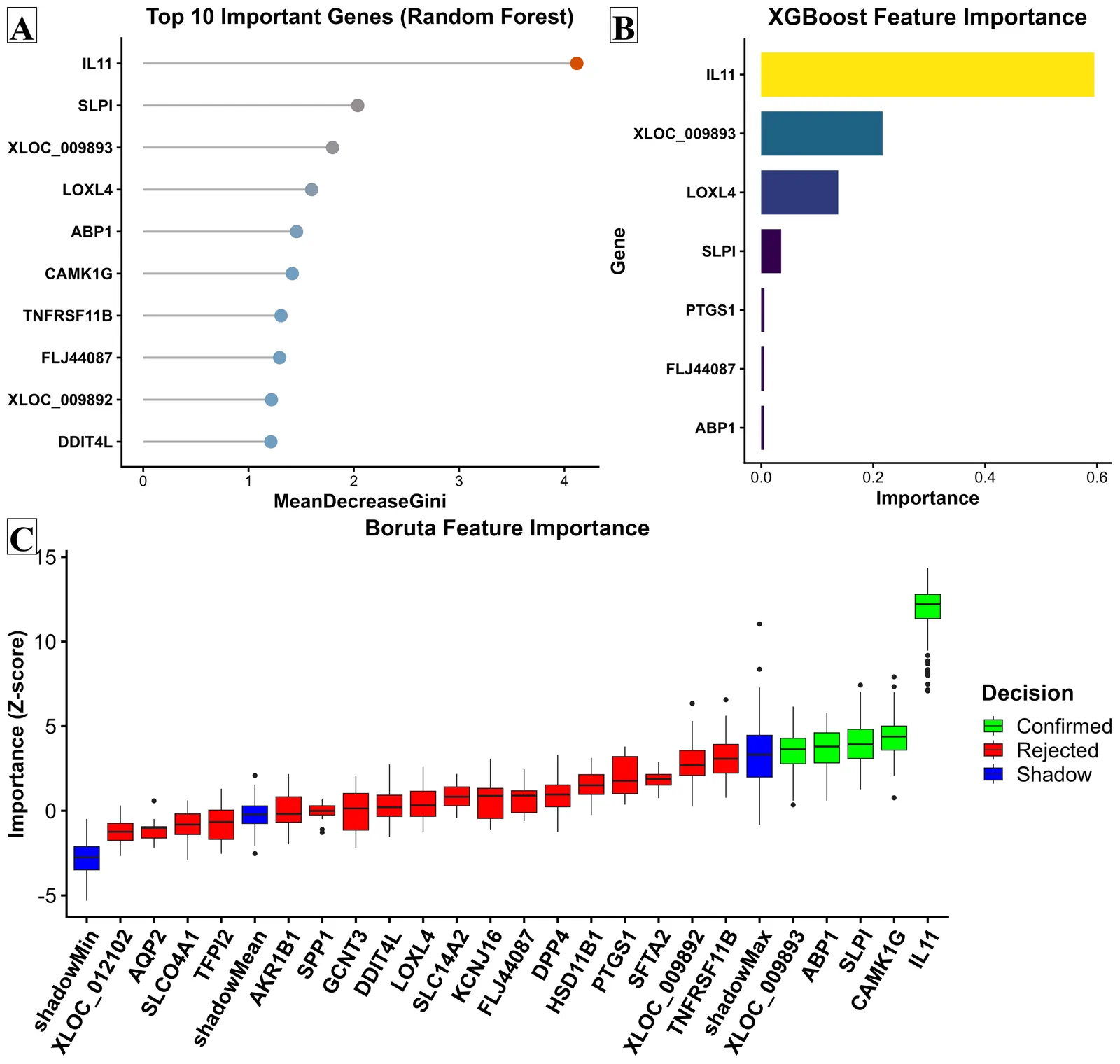
Tree-based and Boruta feature importance. (A) Random-forest variable-importance ranking of the 23 candidate genes. (B) XGBoost feature-importance ranking of the same candidate set. (C) Boruta confirmation results, in which confirmed, tentative, and rejected features are distinguished relative to shadow attributes.

IL11 expression validation and diagnostic performance

In GSE73680, IL11 expression was significantly higher in Case (RP) samples than in Control samples (Wilcoxon P=8.9×10⁻⁵), a finding that is directionally consistent with the upregulation previously reported by Taguchi et al. at the tissue level, by Shen et al. in their comorbidity analysis, and by Li et al. in their combined tissue and serum investigations [6,10,11]. For the penalized logistic model Outcome ~ IL11, the apparent AUC was 0.782 (95% CI 0.663-0.901), the bootstrap-corrected concordance index (C-index) was 0.786, and the patient-blocked out-of-bag (OOB) AUC was 0.756; at the Youden-optimal threshold, accuracy, sensitivity, and specificity were 0.774, 0.621, and 0.909, respectively, indicating high specificity with moderate sensitivity; moreover, the goodness-of-fit test (P=0.406) suggested acceptable agreement between predicted probabilities and observed outcomes. The close proximity between the apparent and OOB AUCs indicates limited overfitting and is consistent with the well-established role of internal validation procedures in quantifying and correcting the optimism of prediction models developed on a single dataset. The resulting nomogram provides a graphical device that maps continuous IL11 expression values onto individualized risk scores, while DCA complements discrimination-based metrics by evaluating the net clinical benefit of the model relative to treat-all and treat-none default strategies across a range of decision thresholds (Figures 8, 9).

**Figure 8:**
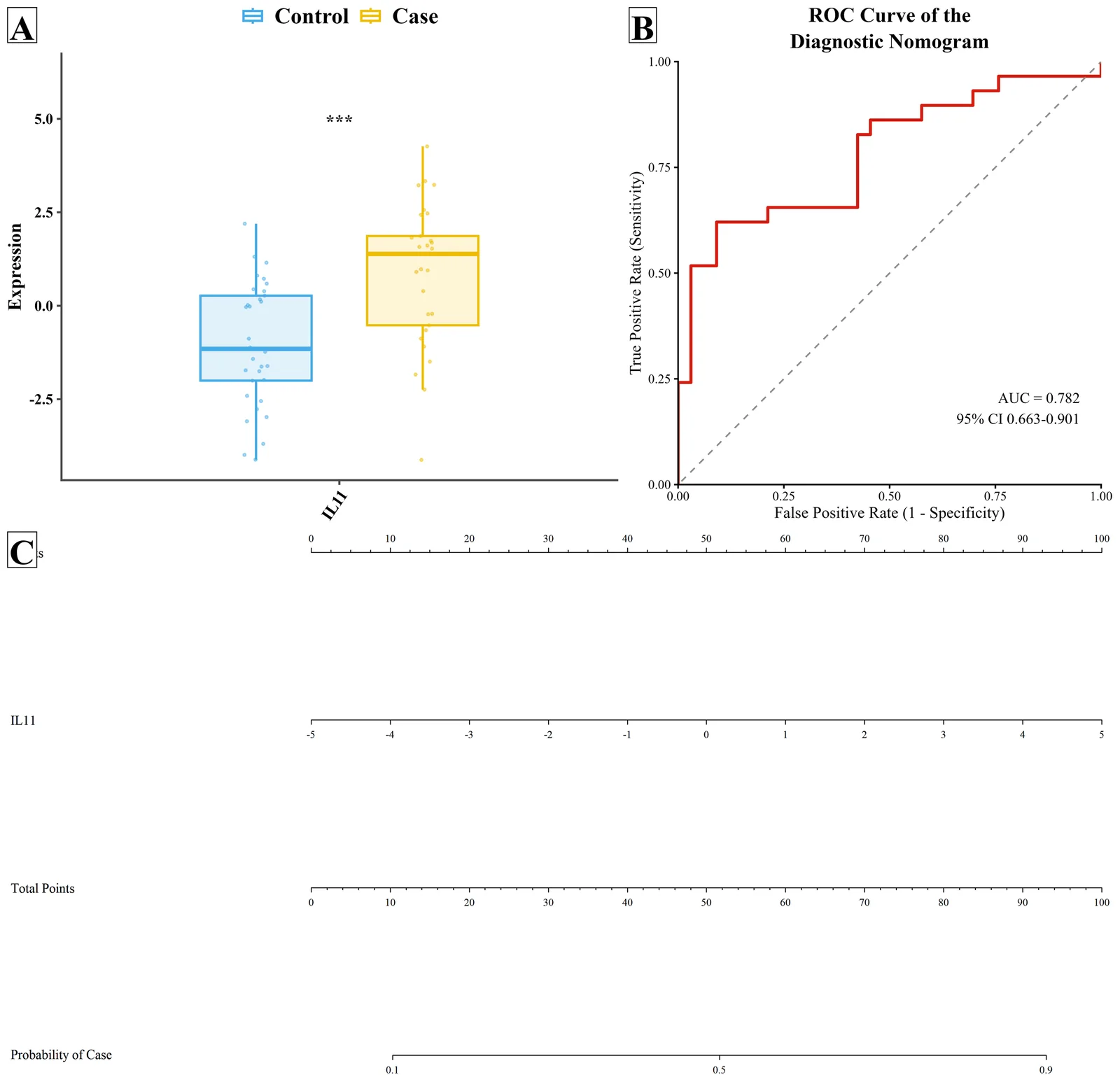
IL11 expression and diagnostic discrimination. (A) Boxplot comparing IL11 expression between Case (RP) and Control samples, with the Wilcoxon rank-sum test indicating a significant between-group difference. (B) ROC curve of the single-gene model, with an apparent AUC of 0.782. (C) Diagnostic nomogram constructed from the penalized logistic model with IL11 as the sole predictor, mapping expression values onto individualized risk scores. ROC: receiver operating characteristic; AUC: area under the curve

**Figure 9:**
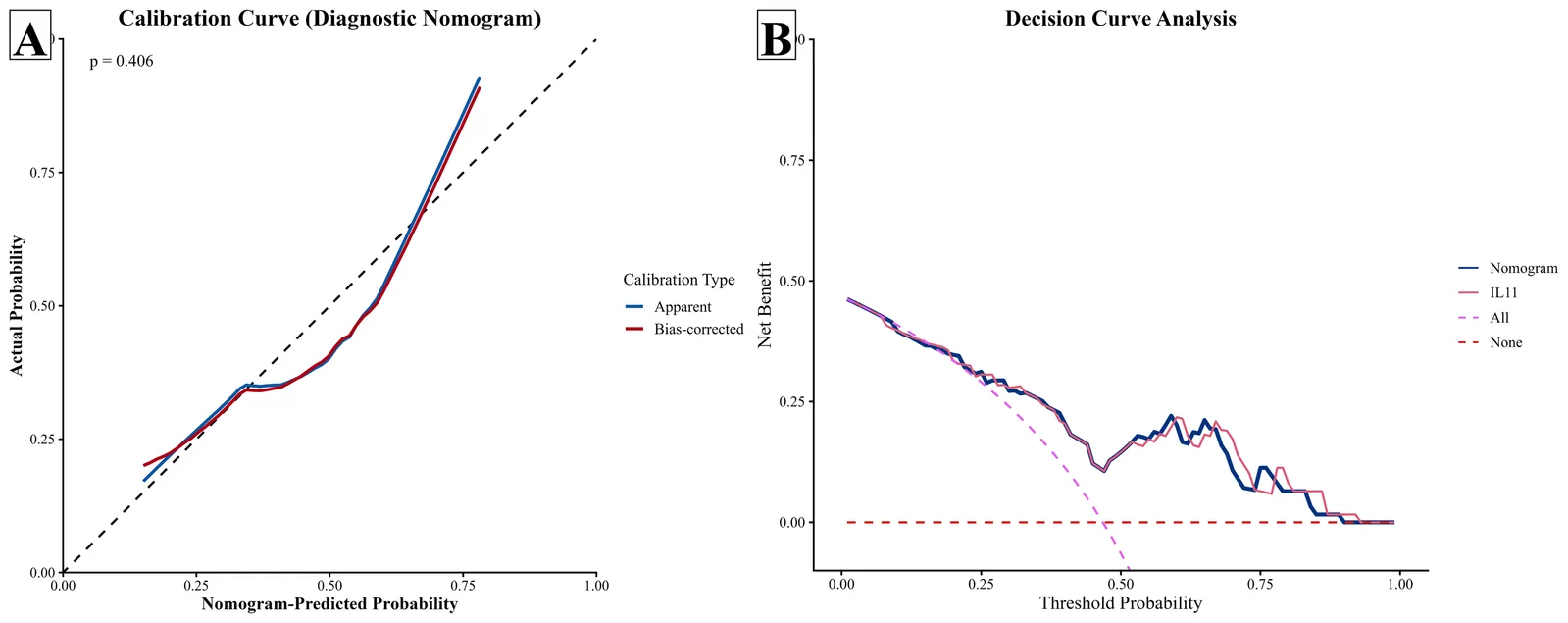
Calibration and decision-curve analysis. (A) Calibration curves showing agreement between predicted probabilities and observed outcomes for both the apparent and the bias-corrected models. (B) Decision-curve analysis (DCA) evaluating the net clinical benefit of the IL11-based model relative to treat-all and treat-none default strategies across decision thresholds.

Immune deconvolution and IL11-correlation GSEA

CIBERSORT deconvolution of the 62 samples revealed substantial sample-to-sample heterogeneity in estimated immune-cell composition, indicating that the immune milieu of papillary tissue varies considerably both within and between study groups. After Wilcoxon rank-sum testing followed by Benjamini-Hochberg (BH) correction for multiple comparisons, none of the 22 estimated cell fractions differed robustly between the Case and Control groups (all adjusted P=1); this result is not fully concordant with some of the cell-level differences reported by Hong et al. [7], a discrepancy that most plausibly reflects the sensitivity of bulk-tissue deconvolution to grouping definitions, preprocessing choices, and multiple-testing strategies rather than a fundamental biological contradiction. Among the Spearman correlations computed between IL11 expression and individual cell fractions, resting memory CD4 T cells showed the strongest positive coefficient (r≈0.325, raw P≈0.010), although this association did not remain significant after BH correction; activated dendritic cells and plasma cells likewise showed only nominal, uncorrected trends. Accordingly, we deliberately refrain from treating single-subset cellular differences as primary conclusions of this study and instead place greater interpretive weight on pathway-level evidence, which aggregates signal across many genes and is generally more robust to deconvolution uncertainty (Figure 10).

**Figure 10:**
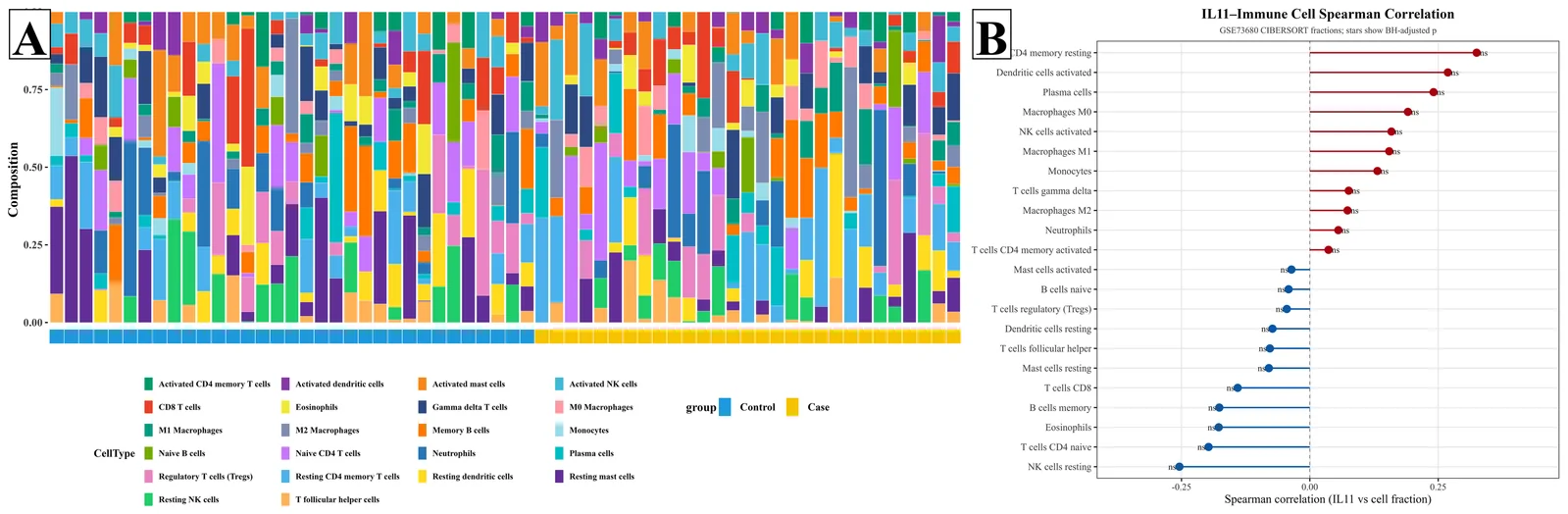
Immune deconvolution and IL11-cell correlations. (A) Stacked bar plot displaying the CIBERSORT-estimated relative fractions of 22 immune-cell types across all samples, illustrating substantial between-sample heterogeneity. (B) Lollipop plot of Spearman correlation coefficients between IL11 expression and the estimated immune-cell fractions, ordered by effect size. CIBERSORT: Cell-type Identification by Estimating Relative Subsets of RNA Transcripts

KEGG GSEA ranked by IL11-genome-wide Spearman correlation coefficients showed that the strongest positive normalized enrichment score (NES) values corresponded to lysosome (NES≈3.40), cell adhesion molecules (CAMs; NES≈3.03), and leukocyte transendothelial migration (NES≈2.99), indicating that genes positively co-expressed with IL11 preferentially cluster within adhesion- and migration-related pathways; conversely, negatively enriched sets included olfactory transduction, linoleic acid metabolism, and retinol metabolism, which likely reflect reciprocal transcriptional programs that are relatively suppressed in high-IL11 states. The enrichment of adhesion and leukocyte-migration pathways fits well with the established immune-matrix interaction framework of RP, in which inflammatory cell recruitment and extracellular matrix remodeling are tightly coupled [4,5]; moreover, IL11 itself appeared among the leading-edge genes of the cytokine-cytokine receptor interaction set, consistent with its identity as a secreted cytokine (Figure 11).

**Figure 11:**
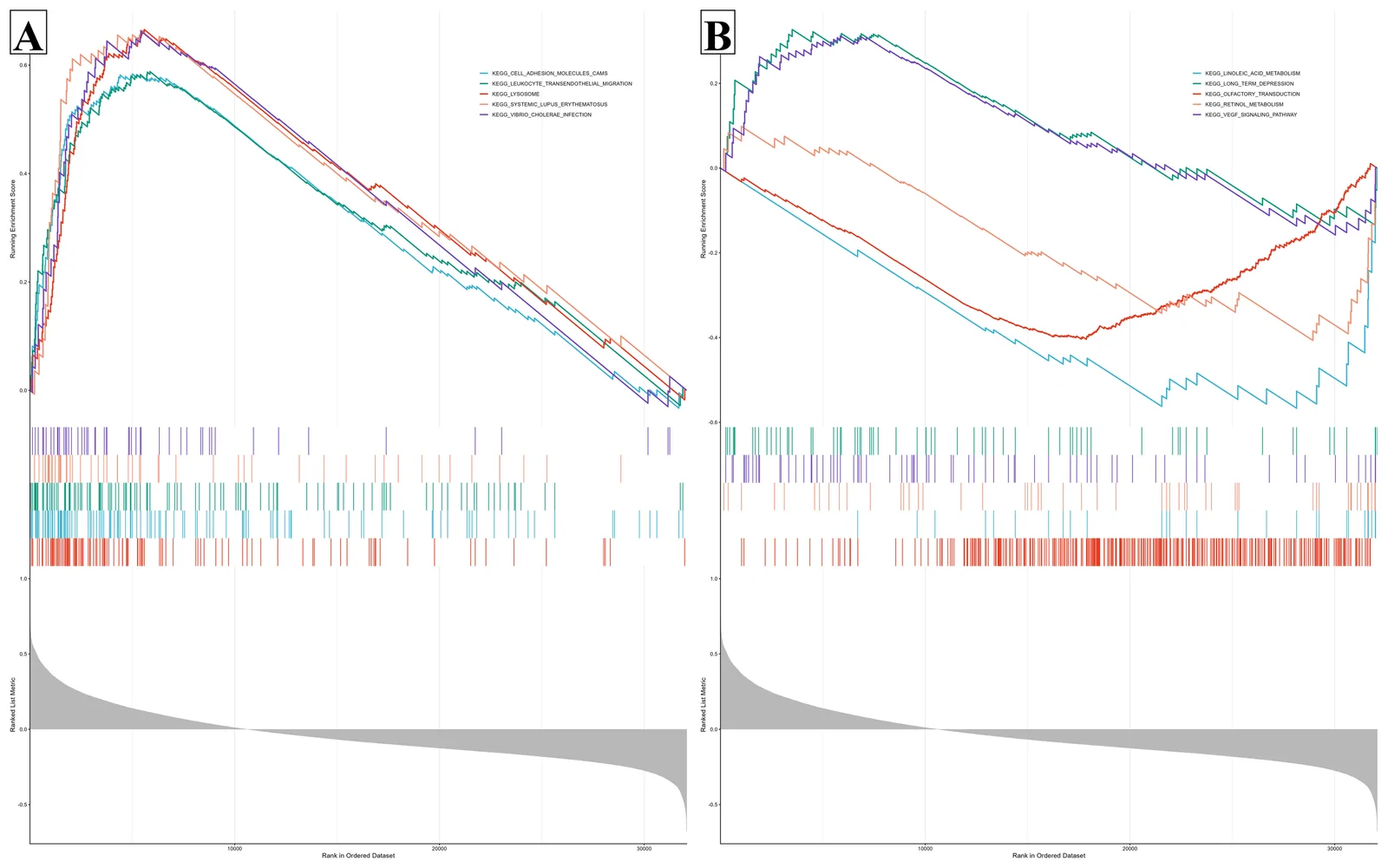
IL11-correlation-ranked KEGG GSEA. (A) Top positively enriched KEGG pathways ranked by normalized enrichment score (NES). (B) Top negatively enriched KEGG pathways ranked by NES. KEGG: Kyoto Encyclopedia of Genes and Genomes; GSEA: gene set enrichment analysis

## Discussion

Using GSE73680, we identified IL11 as the sole five-method consensus gene. This result is supported by upregulation in RP, consistent with the original dataset [6]; membership in the DEG∩WGCNA overlap; PPI links to SPP1 and TNFRSF11B [7]; GSEA pointing to adhesion and leukocyte transendothelial migration, coherent with plaque biology [4,5]; and moderate single-gene discrimination (AUC≈0.78) with internal validation. These findings fit the interstitial inflammation and matrix-remodeling background of RP [4] and independent reports that IL11 is a stone-related candidate at tissue and serum levels [6,10-12]. Taguchi et al. [6] established GSE73680 and first reported IL11 upregulation in human RP. We do not repeat that discovery; we extend secondary analysis by adding WGCNA, five-method consensus, nomogram/calibration/OOB/DCA, and correlation-ranked GSEA, thereby elevating IL11 from a member of an upregulated list to an individually appraised priority target.

Hong et al. [7] used the same 29-versus-33 contrast and ranked SPP1 as the top PPI hub linked to macrophages. In our pipeline, SPP1 remained in the 23-gene overlap and the PPI core but was not selected by all five methods, whereas IL11 was the discriminative consensus gene. The IL11-SPP1-TNFRSF11B links indicate complementary rather than contradictory findings: SPP1 as a network/immune hub and IL11 as a multi-model discriminative feature, jointly pointing to a matrix mineralization-cytokine axis in RP [4,5,7]. Shen et al. [10] addressed shared mechanisms of kidney stones and diabetes, applied three algorithms to 11 comorbidity-overlapping genes, and focused diagnostic appraisal mainly on ROC. Our study uses a pure kidney-stone/RP phenotype, a DEG∩WGCNA candidate pool (23 genes), five algorithms, and deeper diagnostic appraisal (nomogram, calibration, OOB, DCA). Both studies converge on IL11 but differ in question framing and evaluation depth.

Li et al. [11] prioritized IL11 through DEG/PPI modules and provided serum enzyme-linked immunosorbent assay (ELISA) evidence. Our work supplies a more complete tissue-transcriptome screening-diagnosis-mechanism loop but cannot replace their biofluid validation; the two studies are mutually reinforcing [11,12]. Yu et al. [8] emphasized DCN/LUM/P4HA2, and an oxidative-stress re-analysis of the same dataset entered through OS gene sets [9]. Those reports enrich the RP landscape but follow different lead genes. Our narrative centers on IL11 across screening, diagnosis, and GSEA.

Relative to this literature: a pure stone/RP phenotype without diabetes-comorbidity confounding [10]; five-algorithm consensus after DEG∩WGCNA filtering [10]; diagnostic appraisal beyond ROC; and correlation-ranked GSEA plus PPI linkage of IL11 to the SPP1 axis [7]. These points extend the existing evidence chain rather than negate prior discoveries.

No DEG survived strict FDR control, so the exploratory threshold may introduce false positives, although WGCNA filtering and five-method consensus partly constrain chance findings. Diagnostic metrics come from same-cohort internal validation without independent tissue or biofluid cohorts. CIBERSORT is bulk deconvolution with limited resolution. The study is computational and lacks protein-level or functional validation. IL11 perturbation experiments in stone-related models, together with serum-marker work of the kind reported by Li et al. [11], are a logical next step.

## Conclusions

Integrative re-analysis of GSE73680 demonstrated that intersecting exploratory DEGs with a WGCNA trait-related module yields a biologically prioritized pool of 23 candidate genes and that five conceptually distinct algorithms, namely LASSO, SVM-RFE, random forest, XGBoost, and Boruta, uniquely and unanimously converge on IL11 as their sole shared selection. Subsequent validation analyses showed that IL11 is significantly upregulated in RP tissue, that it achieves a single-gene diagnostic AUC of approximately 0.78 with stable internal validation, and that it is physically and functionally connected to SPP1 and TNFRSF11B within the PPI network; in parallel, IL11-correlation-ranked GSEA highlighted coordinated enrichment of lysosome, cell adhesion molecules, and leukocyte transendothelial migration pathways. The present work is most accurately positioned as a methodological consolidation and mechanistic-clue supplementation that strengthens the case for IL11 as a priority kidney-stone-related target; its principal contribution is an actionable, multi-method evidence-based ranking that can directly guide subsequent experimental validation.
